# Remarks on the Influenza

**Published:** 1843-07-22

**Authors:** David H. Tucker

**Affiliations:** Physician to the Philadelphia Dispensary


### REMARKS ON THE INFLUENZA.

BY DAVID H. TUCKER, M. D.

Physician to the Philadelphia Dispensary.

In presenting the following remarks on the subject of the influenza, which is prevailing at present in this city, I beg leave to disclaim any aim at originality, for with the few cases that have come under my care, it would be impossible to draw any just conclusions, either as to the nature of the disease or its treatment. My only aim is, briefly to collect under one head, what has been said on this subject since its first appearance.

The first accounts of the grippe, on which any reliance can be placed, will be found in the year 1580. It then traversed Europe, Asia, and Africa, and the account given of it will be found to correspond very nearly with the opinions of the present day.

Salius Diversus, says, "Almost all were seized with it, no age, sex, or condition was spared—it passed rapidly from one locality to another, commencing in the Winter and continuing during the Spring, Summer, and Autumn, without any regard to the state of the weather, or the geographical character of the country." The same account is given of this epidemic by others. Since this period it has visited Europe, &c. repeatedly, but especially in 1733, in 1743, in 1775, in 1782, in 1830, in 1833, &c. Although the history of the epidemic at these different periods is less accurate than we could have wished, yet it is sufficiently so to convince us that the disease we now have in the United States, is similar to the epidemic described as early as 1580. To avoid being tedious, we shall content ourselves with the above statement and pass to matters of more importance.

*Pathology.*—Authors have differed considerably as to the nature of this disease. We extract briefly from a discussion before the Royal Academy of Medicine, the opinions of several distinguished Pathologists. M. Recamier says, "The grippe of 1837 is not a bronchitis, but a catarrh analogous to eruptive diseases—there is fever, headache, and often an eruption on the skin, redness of the eyes, sore throat, sneezing, cough, &c.," and he adds, "that these symptoms are not only more severe but more difficult of cure than those of an ordinary catarrh." M. Piorry says that the symptoms resemble at first those of eruptive fevers, but soon local symptoms arise, such as congestion of the nares, of the larynx and of the great bronchial tubes; the lungs are not yet affected, the sound being clear and the respiration pure; as the disease advances the smaller bronchia and the lungs become affected, as is indicated by the different sounds. M. Lepelletier considers it "a catarrh with spasm." M. Delorme insists that it should not be classed under the same head as bronchitis or pulmonary catarrh, and for the following reasons: "Bronchitis is under the influence of certain climates, seasons, and localities, and prevails principally in those seasons remarkable for sudden variations in temperature—it attacks those persons predisposed to it, either by old age or infancy, or by a feeble constitution. The grippe, on the contrary, attacks indiscriminately every one; it rages in all climates, in all seasons, and in localities the most different, but always marked by the same characters." Besides, there is considerable difference in the physical signs of the two diseases. And there is in bronchitis, (unless of very long standing,) an absence of that great debility which always accompanies the grippe from its commencement to its termination, and indeed, often continues for some time after the local symptoms have disappeared. The duration of the two diseases differ greatly, for while bronchitis has no definite length and yields to a certain course of treatment, the grippe rarely lasts more than four or five days, and treatment has very little effect in arresting its progress. Dr. Hufeland considers the grippe "a catarrhal fever, accompanied with, and followed by extraordinary depression of the nervous energy, for several weeks after the pyrexia has ceased."

A correspondent of the Med. Chirurg. Rev. states that he, and other physicians, had remarked that, for some time previous to the appearance of the epidemic, there had been a tendency in most febrile complaints to a nervous or adynamic type, and this is in accordance with the history of other epidemics. Dr. Blackiston, of Birmingham, considers the influenza an affection of the nervous system, with its concomitant derangement in the organs of digestion, circulation, &c., commonly known under the name of nervous fever, and accompanied throughout its whole course by irritation of the pulmonary mucous membrane. Dr. Johnson, of the Med. Chir. Rev. says that the disease is always marked by depression and derangement of the nervous system, rather than by inflammatory symptoms of the respiratory apparatus. In one respect, we think, it bears a strong analogy to typhoid fever—in this fever, the glands of Peyer, &c., are constantly affected to a certain extent, at times amounting to ulceration—but no one supposes that the affection of these glands, alone produces the great debility and constitutional disturbance which are always present in this disease; there must be some hidden cause which we cannot discover. So with the grippe, the congestion of the mucous membrane of the air passages will not account for the great nervous prostration. Besides, if this congestion were the only disease, why does it not yield to the ordinary antiphlogistic treatment, as most simple congestions or inflammations do? It may be defined a disease *sui generis*, of a catarrhal nature, marked by an extraordinary tendency to nervous prostration. The local symptoms consist in a congestion of the air passages, often accompanied by rheumatic pains and considerable derangement of the digestive functions.

*Morbid Appearances.*—The disease may be divided into,

1st. Its simplest form.

2d. The suffocating form.

3d. Those cases of influenza complicated with pneumonia.

In the first variety, the mucous membrane of the nares, larynx, and trachea, are in a simple state of congestion. In the second variety the inflammation has extended itself to all the bronchial tubes, and we find these tubes filled with a great quantity of mucous; the mucous membrane is of a uniform red color, slightly tumefied and softened; the lungs are gorged with blood, without softening of their tissue. The third variety is the most fatal. In these cases, in addition to the appearances of the mucous membrane of the bronchial tubes, we have the ordinary hepatization of the lungs, which exists in pneumonia, but what is very singular, the mucous membrane of the bronchia traversing the hepatised portions, is very frequently found covered with a thick false membrane. For these post mortem observations we are indebted to M. Nonat, of Paris, in an article, inserted in vol. xiv. of the "Archives de Médicine," under the head of Grippe. The blood coagulates, but less firmly than in ordinary cases, and its consistence depends upon the inflammatory or adynamic character of the influenza.

*Causes.*—Though some authors consider the grippe contagious, we are disposed to attribute its propagation from one member of a family to another, from one section of country to another, rather to an infected state of the atmosphere, than to a direct contagion. It is this peculiar "constitution of the air," though unappreciable to our senses, which gives rise to cholera, yellow fever, typhus fever, dysentery, &c.

A great number of physicians have kept registers, with a view of ascertaining to what extent this influenza is influenced by the conditions of the atmosphere. Dr. Black, in a paper read before the British Association for the Advancement of Science, says, "contemporaneous with this lowest state of the atmospheric pressure, commenced the full and rapid invasion of the epidemic. As the disease advanced, the temperature fell for seven days, accompanied with rain and snow. The invasion of the epidemic was preceded by, and attended with easterly and southerly winds, while the air was much loaded with moisture." If other registers gave the same result, the conclusion would be easy; but unfortunately they do not. In France in 1830 the epidemic was preceded by very cold weather; whereas, at Geneva it was the reverse. The physicians in France think that the grippe prevails entirely independent of all meteorological changes of the air. Dr. Gluge, of Berlin, in a prize essay, states, that on comparing the registers at different places, it must be evident that the epidemic has prevailed every where, entirely uninfluenced by atmospheric changes. He adds, what is very curious, that prior to the eleventh century its course was from West to East, since then it has been from East to West. Mr. James P. Espy, in a letter to Dr. Hays, suggests that the epidemic influenza may have been caused by the extreme dryness of the air, rather than by its coldness; for that during the month of December, 1831, when the grippe prevailed, he found, "by unexceptionable experiments, that a man of ordinary size exhaled from his lungs every day about 839 grains more than he did each day on the preceding month." In the present epidemic, we see it commencing in the North and passing, without any regard to temperature, towards the South. It has been stated in the Boston papers, that as soon as the British steamers entered the port, the persons on board have become affected with the same epidemic that was prevailing in the city. The same thing has occurred to the "Independence" and "Union," which were stationed off New York during the prevalence of the grippe in that city.

With this want of satisfactory evidence as to the causes which give rise to this epidemic, we must conclude that a certain condition of the air, not appreciable by us, is absolutely necessary to its production. It is true that this explains nothing, but it is at least as intelligible as many of the received opinions as to the origin of other diseases. Intermittent and remittent fevers are said to be produced by malaria, but has any one ever told us what that malaria consists in? So with cholera. Has its origin ever been satisfactorily explained? Every epidemic, probably, has what Sydenham termed its peculiar "constitution of the atmosphere," but what this consists in, is a matter at present beyond our grasp. Sudden changes in the weather, during the prevalence of the influenza, would, it is reasonable to suppose, have some effect in rendering an attack more severe; but every day's experience proves to us that it is not the source of this peculiar epidemic.

*Symptoms.*—The disease is ushered in by great pain over the frontal sinuses, coryza, lachrymation, sore throat, heat and dryness in the fauces, cough dry at first, dyspnœa considerable, anorexia, loss of taste, tongue covered with brown fur, fever sometimes considerable, and great disturbance in the nervous system. Delirium occasionally exists. In some cases we have violent rheumatic pains in the limbs and back. The bowels are sometimes costive, with nausea, vomiting, &c., and in a case recorded by M. Recamier they were found considerably inflamed. Should the disease extend to the bronchia, or be complicated with bronchitis or pneumonia, we will have the rational and physical signs, indicating their presence—but in the simple form, where the phlogosis only extends to the trachea, all the sounds will be formed in a normal state.

*Duration, &c.*—The ordinary duration of simple grippe is from four to eight days, though it may be more, especially when complicated. Dr. Black, in a paper read before the British Association for the Advancement of Science, thinks that persons from twenty to fifty years of age are most susceptible to it—and this accords with Louis' opinion as to typhoid fever. All sexes are equally liable to it.

*Treatment.*—The treatment of the mild form of the disease is very simple. Diet, diaphoretics, and a gentle purgative are all that is necessary. In the severer forms, it will be necessary to cup, blister and bleed, but always bearing in mind the tendency to nervous prostration; and indeed, it is certain that bleeding very often fails to relieve the bronchitis or pneumonia. After the depletion has been sufficiently tried, stimulants may be necessary. We must always be guided by the condition of the patient, for no general rule can be laid down.

